# Hematobiochemical variability and predictors of new-onset and persistent postpartum preeclampsia

**DOI:** 10.1038/s41598-022-07509-5

**Published:** 2022-03-04

**Authors:** Linda Ahenkorah Fondjo, Beatrice Amoah, John Jude Annan, Enoch Appiah Adu-Gyamfi, Evans Adu Asamaoh

**Affiliations:** 1 Department of Molecular Medicine, SMD, KNUST, Kumasi, Ghana; 2 Department of Obstetrics and Gynaecology, SMD/KATH, Kumasi, Ghana

**Keywords:** Biomarkers, Reproductive disorders

## Abstract

Preeclampsia (PE) can occur antepartum or postpartum. When it develops de novo after childbirth, it is termed new-onset postpartum PE (NOPPE). Often, antepartum PE disappears after childbirth; however, in some women it persists after childbirth. This form of PE is termed persistent PE (PPE). Thus, there are two forms of postpartum PE: NOPPE and PPE. The pathogenesis and pathophysiology of these diseases have not been fully characterized, and whether NOPPE and PPE are different or similar pathological conditions remains unexplored. Thus, we aimed to compare the haematological and biochemical characteristics of NOPPE and PPE, predict the occurrence of new-onset PE and identify lifestyles that predispose women to postpartum PE. A total of 130 women comprising 65 normotensive postpartum women, 33 NOPPE and 32 PPE women were recruited for this hospital-based case–control study. The socio-demographic and lifestyle characteristics of the participants were obtained through well-structured questionnaires. Haematological and biochemical indices were measured using automated analysers and ELISA. The prevalence of postpartum PE was 11.9%. Dyslipidaemia (p =  < 0.0001), hypomagnesaemia (p =  < 0.001), elevated serum levels of ALT, AST (p =  < 0.0001), sVCAM-1 (p =  < 0.0001) and sFlt-1 (p =  < 0.0001) were more prevalent and severe in the PPE than in the NOPPE. Sedentary lifestyle was common among both groups of hypertensive women. Elevated ALT and AST were significant predictors of NOPPE. These findings indicate that preeclampsia exists after childbirth in a high percentage of women. NOPPE and PPE are different pathological conditions that require different clinical management. Combined glucose, lipid and liver assessment could be useful in predicting postpartum PE.

## Introduction

Preeclampsia (PE) is a well-recognised disorder associated with adverse maternal and perinatal outcomes. PE can develop antepartum (during pregnancy) or postpartum (after childbirth), and manifests prominently with hypertension and proteinuria^1,2^. In some women PE disappears after childbirth, and in other women it persists after childbirth; whereas in some women, it develops de novo after childbirth. When PE develops during pregnancy, it is termed antepartum or intrapartum PE; and if antepartum PE remains after birth, it is termed persistent PE (PPE). When a normotensive woman develops PE de novo after birth, it is termed new-onset postpartum PE (NOPPE). This usually occurs three to six days after delivery—when most newly delivered mothers would have been discharged—and can remain up to the sixth week after birth. NOPPE and PPE are collectively termed postpartum PE. While some have reported that the prevalence of NOPPE ranges from 0.3 to 27.5% in the US^3,4^, another US study reported postpartum hypertension to be 63.2%^5^ whereas in an African setting of Burkina Faso a prevalence of less than 2% was reported^6^.

Antepartum PE is largely understood as a disorder arising from an abnormal placentation. It is thought that impaired placentation leads to the release of abnormal levels of some angiogenic, anti-angiogenic and vasoactive molecules into maternal circulation, triggering maternal endothelial dysfunction, systemic inflammation, elevated blood pressure and proteinuria^7,8^. Altered maternal serum levels of these molecules have been suggested as potential biomarkers of antepartum PE^7–9^ while elevated serum levels of soluble fms-like tyrosine kinase 1 (sFlt-1) and decreased serum levels of placental growth factor (PlGF), as well as elevated sFlt-1: PlGF, have been validated as biomarkers of this disease^10–13^. Contrary to antepartum PE, the pathogenesis of NOPPE has not been explored; and there are currently no documented biomarkers that can enhance early prediction and diagnosis of this form of PE. Also, the mechanism by which antepartum PE progresses to PPE is unknown. Moreover, the prevalence of postpartum PE (NOPPE and PPE) in sub-Saharan Africa is generally unknown. In high–risk women with postpartum PE, complications such as cardiac failure, cerebrovascular accidents and acute oliguric renal failure, HELLP syndrome have been reported^14^. Yet, specific guidelines for the management of postpartum preeclampsia are not well-defined, which may often result in imprecise diagnoses and inappropriate treatment strategies.

In this study, we hypothesized that the haematological and biochemical characteristics of NOPPE are different from PPE and that women who experience PPE may have altered endothelial function and placental mal-angiogenesis. Thus, we determined the prevalence of postpartum PE in the Ghanaian population, compared the haematological and biochemical characteristics of NOPPE and PPE and identified lifestyle practices that predispose women to these disorders, in an effort to early identify pregnant women at risk of developing NOPPE and PPE.

## Methods

### Study site

This study was conducted at the postnatal clinic of the Komfo Anokye Teaching Hospital (KATH) and the Kumasi Regional Hospital (KRH), both in the Ashanti region of Ghana. KATH, the second largest tertiary hospital in Ghana with over a thousand bed capacity, serves as a major national referral centre that provides health services. KRH, is a primary care hospital in the South-central part of Kumasi, Ghana and provides health services to several communities within the locality. Both facilities manage major obstetric complications from across regions and within community and this makes the recruited study participants a fair sample representing the population of women in Ghana.

### Study design and participants

This hospital-based case–control study recruited a total of 130 women who sought antenatal and postnatal services at the Obstetrics and Gynaecology department of KATH and KRH. They consisted of 65 postpartum normotensives, 33 NOPPE and 32 PPE women, the cases and controls were age-matched. The NOPPE group refers to the women who were normotensive during pregnancy but developed PE after childbirth; while the PPE refers to women with antecedent diagnosis of antenatal PE. The socio-demographic and lifestyle information of the participants were collected through well-structured pre-tested questionnaires. The diagnosis of PE (new-onset and persistent) was done by qualified Obstetrician/Gynaecologist, using the criteria of the American College of Obstetricians and Gynecologists. Thus, PE was defined as elevated systolic blood pressure (≥ 140 mm Hg and/or diastolic blood pressure ≥ 90 mm Hg) and proteinuria (≥ 2 + on urine dipstick); or as hypertension and one, more or all of the following: thrombocytopenia (platelet count < 100,000 per microliter); pulmonary edema; impaired liver function (twice the normal concentration of liver transaminases), new-onset cerebral or visual disturbances; and/ or new renal insufficiency (> 1.1 mg/dl or doubling of serum creatinine).

### Eligibility criteria

For the retrospective study, all women who had given birth within the study period, irrespective of their parity and gravidity were included. For the case–control study, women who had elevated blood pressure within 48 h to 6 weeks after delivery were recruited as cases and normotensive postpartum women were recruited as controls. Both the normotensives and the women with postpartum PE had no history of preconception hypertension, renal disease, type 2 diabetes mellitus, gestational diabetes, HIV/AIDS.

### Blood pressure measurement

The blood pressure of each participant was measured in the morning prior to venous blood sample collection. Each participant was asked to sit down comfortably, extend the left arm on a table and then relax for 5 min. A trained personnel used a mercury sphygmomanometer (Accoson, England) and a stethoscope to measure the blood pressure of all participants in accordance with standard guidelines^15^. Blood pressure was measured twice for each participant 6–15 min apart. The procedure was repeated for each participant, and mean values of the duplicate measurements were recorded to the nearest 2.0 mmHg.

### Urine sample collection and estimation of proteinuria

The participants provided 10 ml of freshly voided early morning urine in clean leak-proof containers. Proteinuria was assessed using semi-quantitative colour scale on the urine reagent dipstick (URIT 2V^PG^ Medical electronic Co., Ltd. China). Proteinuria was defined as the presence of urinary protein ≥ 2 + on urine dipstick.

### Blood sample collection

Ten (10) millilitres of venous blood sample were collected in the morning from each participant after an 8–12 h overnight fast under aseptic conditions. Five millilitres (5.0 ml) of the blood was dispensed into serum separator tubes for biochemical analysis, another 2.5 ml was dispensed potassium oxalate tube for glucose determination whiles the remaining 2.5 ml of the blood was dispensed into the sample tubes containing Potassium Ethylene Diamine Tetra-acetic acid anticoagulant (K_3_EDTA). The blood sample in the K_3_EDTA tube was used for haematological analysis while the serum was separated and stored in Eppendorf tubes at − 80 °C for biochemical analysis.

### Measurement of haematological parameters

The 2.5 ml blood in the K_3_EDTA sample tube was mixed thoroughly and used for the full blood count determination using an automated haematological analyser (Sysmex Automated Haematology Analyser, Kobe, Japan, XP-300, Model No: AC580857). The hemoglobin (HB) level, red blood cell (RBC) count, white blood cell (WBC) count, mean cell volume (MCV) level, neutrophil (NEU) count, lymphocyte (LYM) count and platelets count of the study participants were recorded.

### Biochemical assay

The five (5) ml venous blood dispensed into serum separator tubes was allowed to stand for about 15–30 min to clot and then centrifuged (Nüve NF 200, Germany) at 2000 × *g* for 15 min. Serum was aliquoted under sterile conditions and stored at − 80 °C (Thermo Scientific™ Revco™ UxF − Ultra-Low Temperature Freezers, USA) until assayed. Serum levels of sVCAM-1 and sFlt-1 were measured in duplicates using commercially available ELISA kits from R&D System Inc. (Minneapolis, MN USA). The optical density was measured at 450 nm using a microplate ELISA reader (Mindray MR-96A; Shenzhen Mindray Biomedical electronics Co., Ltd, China). The serum levels of total cholesterol (TC), low-density lipoprotein (LDL) cholesterol, high-density lipoprotein (HDL) cholesterol, triglyceride (TG), alanine transaminase (ALT), aspartate transaminase (AST), sodium ions and potassium ions were measured with automated chemistry analyser (LE scientific, Horizon 850, China). The blood dispensed into the potassium oxalate tube was used to determine Fasting Blood Glucose (FBG) using the glucose oxidase/peroxidase method.

### Data for prevalence analysis

Data for the prevalence study was abstracted from the Records Department of the Kumasi Regional Hospital (KRH). All pregnant women who presented to the hospital with and without PE during the 2-year under review (2017 and 2018), were included in the study and their information retrieved, documented and analysed.

### Ethical consideration

The study protocol was approved by the Committee on Human Research Publications and Ethics (CHRPE) (CHRPE/AP/037/18), School of Medical Sciences, Kwame Nkrumah University of Science & Technology (KNUST) and the Research and Development Committee of KATH and the KRH. The study was conducted in accordance with the declaration of Helsinki. Each participant gave their written informed consent after the aim of the study had been explained to them.

### Statistical analysis

Statistical analysis was performed using SPSS and Graphpad Prism® version 5.0 (Graph Pad Software Inc., Los Angeles). Shapiro–Wilk normality test was used to test the data for a normal distribution. Continuous data were reported as mean ± standard deviation (SD) or median interquartile range where appropriate. Categorical data were presented as frequency (percentage). Association between categorical variables was determined using the Chi-square or Fischer’s Exact test. The one-way ANOVA followed by the Tukey multiple comparison test or the Kruskal Wallis test followed by Dunnet C multiple comparison test were used to compare the differences in parameters between the three groups where appropriate. The univariate and multivariate logistic regression analysis was used to determine the predictors of NOPPE. The area under the curve (AUC) was used to determine the accuracy of haematological and biochemical markers for distinguishing women who are at risk of developing NOPPE. Statistical significance was accepted at p < 0.05 for all comparisons.

## Results

### Determination of the prevalence of postpartum PE (NOPPE and PPE)

At the time of data collection, records of women presenting with postpartum PE at KATH was unavailable. Due to this, the prevalence was obtained from only KRH. During the two-year period under review (2017 and 2018), a total of 3746 deliveries was recorded. Out of the total, 445 postpartum PE cases were recorded. The prevalence of postpartum PE was 11.9%. The prevalence was computed as follows:

$$= \frac{445}{3746} \times 100\%=11.9\%$$

### Socio-demographic characteristics of the study participants

Table 1 displays the socio-demographic characteristics of the participants. Most of the normotensives were within the 20–29 years age range, whereas most of the NOPPE and PPE women were within the 30–39 years age range. Nonetheless, age distribution did not differ across the study groups (p > 0.05). The participants were comparable in terms of educational status, religion, marital status, employment status, residence and ethnicity (p > 0.05).

**Table 1:** Socio-demographic characteristics of the participants.

Parameters	Normotensives (n = 65)	NOPPE (n = 33)	PPE (n = 32)	p-value
**Age (Mean ± SD)**	28.77 ± 6.37	29.70 ± 6.62	31.56 ± 8.07	0.176
**Age categories**				0.062
≤ 19	4(6.2)	2(6.1)	2 (6.3)	
20–29	33(50.8)	12(36.4)	10(31.3)	
30–39	27(41.5)	17(51.5)	14(43.8)	
40–49	1(1.5)	2(6.1)	6(18.8)	
**Education**				0.561
None	7(10.8)	5(15.2)	7(21.9)	
Basic	37(56.9)	17(51.5)	13(40.6)	
Secondary	16(24.6)	6(18.2)	9(28.1)	
Tertiary	5(7.7)	5(15.2)	3(9.4)	
**Religion**				0.547
Christianity	58(89.2)	28(84.8)	26(81.3)	
Islam	7(10.8)	5(15.2)	6(18.8)	
**Marital status**				0.315
Single	18(27.7)	13(39.4)	6(18.8)	
Married	46(70.8)	19(57.6)	26(81.3)	
Divorced	1(1.5)	1(3.0)	0(0.0)	
**Employment**				0.619
Self-employed	45(69.2)	18(54.5)	19(59.4)	
Civil servant	8(12.3)	6(18.2)	4(12.5)	
Unemployed	12(18.5)	9(27.3)	9(28.1)	
**Residence**				0.859
Urban	39(60.0)	20(60.6)	21(65.6)	
Rural	26(40.0)	13(39.4)	11(34.4)	
**Ethnicity**				0.408
Akan	45 (69.2)	27 (81.8)	23 (71.9)	
Non-Akan	20 (30.8)	6 (18.2)	9 (28.1)	

Values are presented as frequency (proportion) or Mean ± SD. Chi-square or Fischer exact test was used to compare between groups. A p-value < 0.05 indicated significant differences across and between groups.

### Lifestyle characteristics of the participants

Table 2 shows the lifestyle characteristics of the three groups of women studied. The proportion of women who were engaged in scheduled physical activities differed across the groups (p < 0.0001). A significant proportion of the normotensives were into regular planned exercises compared to the NOPPE and PPE women (p < 0.05). Conversely, significant proportions of the NOPPE and PPE women were engaged in little and irregular physical activities compared to the normotensives (p < 0.05). These proportions did not differ between the NOPPE and PPE women (p > 0.05). The NOPPE and PPE women were comparable in terms of their engagement in physical activities (p > 0.05). The proportions of the participants with regards to alcohol use, smoking status, parity and change of sexual partner were not different (p > 0.05). The proportion of women who had a family history of hypertension differed among the participants (p < 0.001). Compared to the normotensives, significant proportions of the NOPPE and PPE women had a family history of hypertension (p < 0.05); but between the NOPPE and the PPE women, these proportions were comparable (p > 0.05).

**Table 2:** Lifestyle characteristics of the study participants.

Parameters	Normotensives (n = 65) (a)	NOPPE (n = 33) (b)	PPE (n = 32) (c)	*p* value	Sign. pairs
**Physical activity**				< 0.0001	
Irregular physical activities	16 (24.6)	17 (51.5)	16 (50.0)		a&b;a&c
Little exercise	4 (6.2)	14 (42.4)	14 (43.8)		a&b;a&c
Regular exercise	45 (69.2)	2 (6.1)	2 (6.3)		a&b;a&c
**Alcohol use**				0.194	
No	63 (96.9)	30 (90.9)	28 (87.5)		
Yes	2 (3.1)	3 (9.1)	4 (12.5)		
**Smoking status**				0.362	
No	65 (100.0)	32 (97.0)	31 (96.9)		
Yes	0 (0.0)	1 (3.0)	1 (3.1)		
**Change of partner**				0.072	
No	58 (89.2)	25 (75.8)	23 (71.9)		
Yes	7 (10.8)	8 (24.2)	9 (28.1)		
**Parity**				0.114	
Para 1	13 (20.0)	10 (30.3)	10 (31.3)		
Para 2	19 (29.2)	4 (12.1)	3 (9.4)		
Multiparity	33 (50.8)	19 (57.6)	19 (59.4)		
**Family history of hypertension**				< 0.001	
Yes	11(16.9)	21(63.6)	16(50.0)		a&b;a&c
No	54(83.1)	12(36.4)	16(50.0)		a&b;a&c

Values are presented as frequency (proportion). Chi-square or Fischer exact test was used to compare between groups. A p-value < 0.05 indicated significant differences across and between groups, Sign. Pairs:significant pairs.

Table 3 shows the anthropometric, haematological and biochemical characteristics of the three groups of women. Mean BMI differed across the groups, with the NOPPE women having a higher mean BMI than that of the PPE women (p < 0.05). The average weight of the babies similarly differed across the groups (p < 0.0001). The mean haemoglobin count was higher in the normotensive women than in the PPE women (p = 0.001) but comparable between the NOPPE and PPE women (p > 0.05). The mean RBC count was higher in the normotensive women than in the NOPPE and PPE women (p = 0.002), but similar between the NOPPE and PPE women (p > 0.001). FBG levels were lower in the normotensives than in the NOPPE and PPE women (p < 0.0001) but comparable between the NOPPE and PPE women (p > 0.05)**.** TC, TG, AST and ALT levels were lower in the normotensives than in the NOPPE and PPE women (p < 0.05) and lower in the NOPPE women than in the PPE women (p < 0.05). LDL-C levels differed across the groups but were not significantly different between any of the pairs (p > 0.05). The levels of magnesium were significantly higher in the normotensives than in the NOPPE and PPE women (P < 0.05), and lower in the NOPPE women compared to the PPE women (p < 0.05). The other parameters did not differ among the three groups (p > 0.05).

**Table 3:** Anthropometric, haematological and biochemical profile of the study participants.

Parameters	Controls	Cases		*p* value	Sign. pairs
	Normotensives (n = 65) (a)	NOPPE (n = 33) (b)	PPE (n = 32) (c)		
**Anthropometric profile**					
Maternal BMI	26.12 ± 4.22	27.11 ± 5.63	24.28 ± 3.89	0.041	b&c
Weight of Baby	3.1 (2.8–3.7)	3.5 (2.75–3.8)	2.55 (2.02–3.05)	< 0.0001	a&b;a&c;b&c
**Haematological profile**					
Haemoglobin	12.30 ± 1.53	11.61 ± 2.16	10.71 ± 2.15	0.001	a&c
RBC	4.16 ± 0.0.67	3.95 ± 0.92	3.55 ± 0.79	0.002	a&b; a&c
WBC	7.02 ± 2.94	7.92 ± 3.64	8.30 ± 3.65	0.157	
Lymphocytes	1.87 ± 0.64	2.11 ± 0.81	2.15 ± 1.19	0.233	
Neutrophils	4.33 ± 2.82	5.04 ± 3.26	5.48 ± 3.10	0.185	
MCV	95.21 ± 8.82	94.32 ± 10.07	93.29 ± 8.90	0.620	
Platelet	243.35 ± 85.00	219.27 ± 96.84	212.41 ± 144.59	0.324	
**Biochemical profile**					
FBG	4.79 ± 1.28	5.71 ± 1.62	6.02 ± 1.60	< 0.0001	a&b;a&c
Total Cholesterol (TC)	4.37 ± 0.86	5.38 ± 1.09	5.91 ± 0.95	< 0.0001	a&b;a&c;b&c
Triglycerides (TG)	1.30 ± 0.32	1.68 ± 0.63	2.27 ± 1.18	< 0.0001	a&b;a&c;b&c
HDL-C	1.11 ± 0.53	1.16 ± 0.52	1.10 ± 0.63	0.902	
LDL-C	2.95 ± 0.97	3.40 ± 1.11	3.52 ± 1.52	0.047	
Sodium	137.46 ± 12.17	137.66 ± 14.46	138.51 ± 18.78	0.944	
Potassium	3.76 ± 1.06	4.14 ± 1.66	4.14 ± 1.74	0.312	
Magnesium	0.84 ± 0.13	0.66 ± 0.10	0.69 ± 0.1	< 0.001	a&b;a&c;b&c
AST	28.04 ± 8.28	62.63 ± 36.48	64.81 ± 29.02	< 0.0001	a&b;a&c;b&c
ALT	27.4(22.4–33.3)	73.9(55.6–118.9)	70.1(60.7–92.7)	< 0.0001	a&b;a&c;b&c

Values are presented as Mean ± SD. One way ANOVA followed by Tukey Post Hoc multiple comparisons was used to compare between groups. A p-value < 0.05 indicated significant differences across and between groups.

*AST* Aspartate transaminase, *ALT* Alanine transaminase, *RBC* Red blood cell, *WBC* White blood cell, *MCV* Mean corpuscular volume, *FBG* Fasting blood glucose, *HDL-C* High density lipoprotein-cholesterol, *LDL-C* Low density lipoprotein-cholesterol, *Sign. Pairs* significant pairs.

### The prevalence of haematological and metabolic disorders among the participants

Table 4 shows the prevalence of haematological and metabolic disorders among the study participants. The proportion of low baby weight differed across the groups (p < 0.001), with the low baby weight being highly prevalent among the PPE women than among the NOPPE and normotensive women (p < 0.05). However, maternal bodyweight was comparable across the groups (p > 0.05). Also, the prevalence of anaemia and low RBC count were higher among the PPE women compared to the NOPPE women (p < 0.05) and normotensive women (p < 0.05). Thrombocytopenia was highly prevalent among the NOPPE and PPE women compared to the normotensive controls. Hypercholesterolemia and hypertriglyceridemia were more common with the PPE women compared to the NOPPE and normotensive women (p < 0.05). Also, high AST and high ALT were more prevalent with the NOPPE and PPE women than with the normotensive controls (p < 0.05).

**Table 4:** The prevalence of haematological and metabolic disorders among the participants.

Parameters	Controls	Cases		*p* value	Sign. pairs
	Normotensives (n = 65) (a)	NOPPE (n = 33) (b)	PPE (n = 32) (c)		
**Anthropometric profile**					
Low baby weight	5 (7.8)	3 (10.0)	11 (36.7)	0.001	a&c; b&c
Maternal overweight	25 (38.5)	11 (33.3)	10 (31.3)	0.752	
Obesity class one	9 (13.8)	7 (21.2)	1 (3.1)	0.093	
Obesity class two	2 (3.1)	3 (9.1)	1 (3.1)	0.366	
**Haematological profile**					
Anaemia	14 (21.5)	12 (36.4)	21 (65.6)	< 0001	a&c;b&c
Low RBC count	18 (27.7)	13 (39.4)	22 (68.8)	0.001	a&c; b&c
Leucocytosis	5 (7.7)	6 (18.2)	6 (18.8)	0.190	
Lymphocytosis	0 (0.0)	0 (0.0)	1 (3.1)	0.214	
Neutrophilia	6 (9.2)	7 (21.2)	8 (25.0)	0.092	
Thrombocytopenia	7 (10.8)	10 (30.3)	12 (37.5)	0.005	a&b;a&c
**Biochemical profile**					
Hyperglycaemia	12 (18.5)	11 (33.3)	17 (53.1)	0.002	a&c; a&c
Hypercholesterolemia	2 (3.1)	8 (24.2)	8 (25.0)	0.002	a&b;a&c
Hypertriglyceridaemia	4 (6.2)	13 (39.4)	20 (62.5)	< 0.0001	a&b;a&c
Low HDL	31 (51.7)	13 (43.3)	19 (63.3)	0.295	
High LDL	28 (43.1)	20 (60.6)	16 (50.0)	0.259	
High AST	0 (0.0)	18(54.5)	20(62.5)	< 0.0001	a&b;a&c
High ALT	1(1.5)	26(78.8)	26(81.3)	< 0.0001	a&b;a&c

Values are presented as frequency (proportion). Chi-square or Fischer exact test was used to compare variables between groups. A p-value < 0.05 indicated significant differences across and between groups.

*RBC* Red blood cell, *HDL* High density lipoprotein, *LDL* low density lipoprotein, *AST* Aspartate transaminase, *ALT* Alanine transaminase.

### Univariate and multivariate analysis of haematological and biochemical factors predictive of NOPPE

Regression analysis of factors associated with NOPPE is presented in Table 5. In the univariate model, thrombocytopenia was significantly associated with an increase in the likelihood of developing NOPPE [OR = 3.60 (1.22–10.61), p-value = 0.020] but not in the multivariate model. Hypercholesterolemia increased the likelihood of NOPPE by 10.08 times (p-value = 0.005) in the univariate model and 32.89 times (p-value = 0.022) in the multivariate model. Hypertriglyceridemia increased the occurrence of NOPPE by 9.91 times (p-value < 0.001) in the univariate model and 18.119 times (p-value = 0.032) in the multivariate model. AST and ALT were associated with increased odds of NOPPE (p-value < 0.001) in the univariate model. ALT increased the odds of developing NOPPE in the multivariate model by 147.83 times (p-value < 0.001). However, AST effect was insignificant in the multivariate model (p-value = 0.120).

**Table 5:** Univariate and multivariate analysis of haematological and biochemical factors predictive of NOPPE.

Variables	Univariate model			Multivariate model	
	New onset	p-value		New onset	p-value
**Haematological profile**					
Anaemia	2.08 (0.83–5.24)	0.120		–	–
Low RBC count	1.70 (0.70–4.11)	0.241		–	–
Thrombocytopenia	3.60 (1.22–10.61)	0.020		8.89(0.502–157.59)	0.136
**Biochemical profile**					
Hyperglycaemia	2.2 (0.85–5.75)	0.105		–	–
Hypercholesterolemia	10.08 (2.00–50.80)	0.005		32.89(1.65–655.71)	0.022
Hypertriglyceridemia	9.91 (2.90–33.89)	< 0.001		18.119(1.29–254.34)	0.032
High AST levels	76.80 (9.49–621.35)	< 0.001		10.67(0.54–210.36)	0.120
High ALT levels	117.0 (22.78–601.07)	< 0.001		147.83(12.28–1779.83)	< 0.001

### Criteria for distinguishing individuals at risk of developing NOPPE

Table 6 shows the haematological and biochemical criteria for distinguishing individuals at risk for NOPPE. At an optimal threshold of > 38.3 and > 39.2U/L for ALT and AST respectively, AUC for ALT and AST [AUC = 0.99(0.98–0.99)] and [AUC = 0.98(0.91–1.00)], respectively, indicated significantly a best diagnostic profile for predicting the occurrence of NOPPE (p-value < 0.0001). TC, TG, FBG, LDL and sFlt-1 levels were significantly associated with predicting the occurrence of NOPPE (p-value < 0.05). AST and ALT levels had the best diagnostic consistency in predicting the occurrence of NOPPE (9.1 vrs 9.5), followed by TG (Kappa = 2.9), LDL (Kappa = 3.1), FBG (Kappa = 1.9) and sFlt-1 (Kappa = 1.7) levels.

**Table 6:** Haematological and biochemical criteria for distinguishing women who are at risk of developing NOPPE.

Test variables	AUC (95% CI)	Sensitivity	Specificity	Cut-off	p-value	Kappa
Platelet	0.58 (0.46–0.71)	0.45	0.82	< 173.0	0.198	0.283
FBG	0.64 (0.53–0.75)	0.85	0.38	> 4.2	0.010	0.185
TC	0.79 (0.69–0.89)	0.64	0.92	> 5.21	< 0.0001	0.590
TG	0.69 (0.58–0.81)	0.55	0.80	> 1.6	0.001	0.290
LDL	0.62 (0.50–0.75)	0.72	0.86	> 3.82	0.049	0.309
AST	0.95 (0.91–1.00)	0.91	0.98	> 39.2	< 0.0001	0.907
ALT	0.99 (0.98–0.99)	0.97	0.98	> 38.3	< 0.0001	0.954

*FBG* fasting blood glucose, *TC* total cholesterol, *TG* triglyceride, *LDL* low density lipoprotein, *AST* Aspartate transaminase, *ALT* Alanine transaminase.

## Discussion

We evaluated the prevalence of postpartum PE, determined the haematological and biochemical variations between NOPPE and PPE and evaluated the predictive factors for developing de novo PE postpartum. In this study, we report a high crude prevalence of 11. 9% for postpartum PE among our study population, a finding in contrast with reports by Zamane et al.^6^ who reported a lower prevalence for a study conducted in another African setting. The high prevalence in this study could be attributed to low knowledge about postpartum PE and the reduced monitoring of women and their blood pressures during the postpartum period. In an earlier study, we reported that most women irrespective of their level of education or parity had low knowledge about PE^16^, this finding re-echoes that little is known about preeclampsia and postpartum preeclampsia especially among the general populace. As such, after delivery, most women seldom have their blood pressures checked or monitored until after one week or two weeks postpartum when their new born babies are given their first immunisation. Very often, all the attention is given to the new born baby, with very little to the mother. Thus, women with asymptomatic hypertension frequently go unnoticed. Also, during the postpartum period, women who develop symptomatic hypertension are usually managed at the outpatient department but not at the postnatal clinic, especially when the baby is not sick. This practice is common in many hospitals, and further limits close monitoring of women in the postpartum period. Clearly, our finding of high prevalence of postpartum PE emphasizes the fact that delivery of the placenta does not discount the possibility for PE; as such, continuous close monitoring of mothers in the postpartum period is paramount.

The finding of higher prevalence of sedentary lifestyle among the NOPPE and PPE women calls for an intensified education on the benefits of exercise in pregnancy (Table 2). Physical activity during pregnancy is highly recommended. It has been reported that exercise is associated with 41% decreased odds of developing PE^17^. Frequent periods of sedentary life decrease the rate of skeletal muscle contractions, leading to a reduction in lipoprotein lipase activity, a decrease in the clearance of TGs and glucose load, and a decrease in the rate of glucose-stimulated insulin secretion^18^. Consequently, chronic dyslipidaemia and hyperglycaemia can emerge and increase the risk of developing metabolic syndrome^19^ and cardiovascular disorders^20,21^ including PE. This is evident in the observed higher TC and TG levels in the PPE women than in the NOPPE women and normotensives, and higher in the NOPPE women than in the normotensives (Table 3). It is noteworthy that, these observations were independent of maternal BMI since the PPE women had the least mean BMI (Table 3). These findings indicate that dyslipidaemia is more characteristic of PPE than NOPPE but predicts an increased risk for NOPPE, (Table 5) and that regular exercise during pregnancy could minimise the risk of developing NOPPE and PPE.

Anaemia is associated with hypertension^22^, renal dysfunction^23,24^ and inflammation ^25^ and hence a typical feature of PE. In this study, while we observed that the prevalence of anaemia and low RBC count was higher among the NOPPE and PPE compared to the controls; difference between the two hypertensive women were comparable, suggesting that anaemia though evident in NOPPE and PPE may not differ in severity between the two clinical conditions (Tables 3, 4). The impairment in renal function associated with PE may have altered erythropoiesis leading to the anaemia in these women. Thrombocytopenia is a common alteration in the coagulation system in antepartum PE^26,27^. In this study, our reports indicate that thrombocytopenia could be a symptom of both NOPPE and PPE, but may not be reliable to distinguish between the two conditions due to the fact that the platelet count was not different across and within the three groups of women (Table 3), and prevalence was not different between the NOPPE and PPE women (Table 4). Conversely, we found hypomagnesaemia to be more pronounced in the NOPPE than in the PPE women (Table 3). Several studies have reported an association between low serum levels of magnesium in PE. Magnesium levels correlate negatively with blood pressure while magnesium supplementation helps in the reduction of blood pressure^28^.

Elevated of sFlt-1 is evident in PE placenta, and inhibits its angiogenesis^29,30^. The excessive inhibition of placental angiogenesis contributes to placental hypoxia and reperfusion injuries which increase oxidative stress within the placenta, triggering antepartum PE^31^. Several epidemiological and experimental studies have reported that the maternal signs and symptoms of antepartum PE are partly induced by elevated serum levels of sFlt-1^30,32–35^. sFlt-1 blocks the receptor-binding domains of vascular endothelial growth factor (VEGF) and placental growth factor (PGF), causing endothelial dysfunction^36^ and when widespread culminates into the hypertension, proteinuria and multisystem damage characteristic of antepartum PE^37^. In this study, the levels of sFlt-1 were not different between the NOPPE women and the normotensive controls, but significantly lower in the NOPPE compared to the PPE women (Fig. 2). It has been reported that sFlt-1 has a shorter half-life, and that its levels rapidly fall after delivery of the placenta, associating strongly with the resolution of maternal PE symptoms within 48 to 72 h^38–40^. This suggests that the placenta is the major source of circulating sFlt-1 in antepartum PE. The elevated levels of sFlt-1 in PPE may explain the persistent PE and its companying signs and symptoms in this category of women. Furthermore, low baby weight was more prevalent among the PPE than in the NOPPE and normotensive women (Table 4), suggesting that PPE predominates with a pre-existing abnormal placentation which affects the physiological interaction between the mother and the foetus, thus depriving the foetus of the needed nutrients and hence restricting its growth rate. The high sFlt-1 in spite of its short half-life in the PPE women may suggest the secretion of this biomarker from non-placental sources, such as peripheral blood mononuclear cells, into the blood.

**Figure 1:**
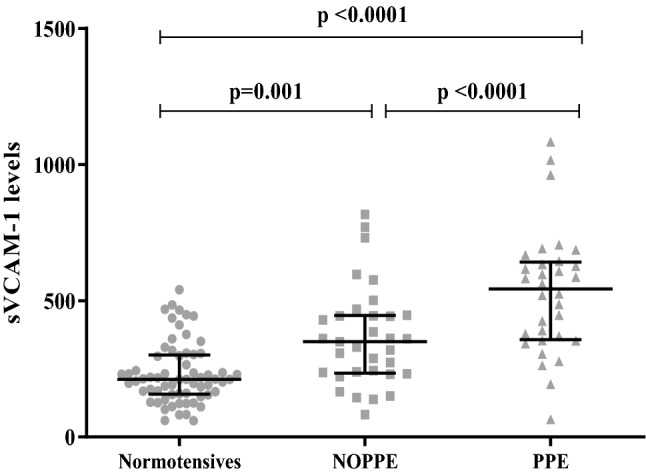
Serum levels of soluble vascular cell adhesion molecule (sVCAM-1) among the study participants. The levels of sVCAM-1 differed among the participants. The NOPPE and PPE women had elevated levels of sVCAM-1, compared to the normotensive controls. Also, the levels were different between the two groups of PE women, with the PPE women exhibiting higher levels of sVCAM-1 than the NOPPE women.

**Figure 2:**
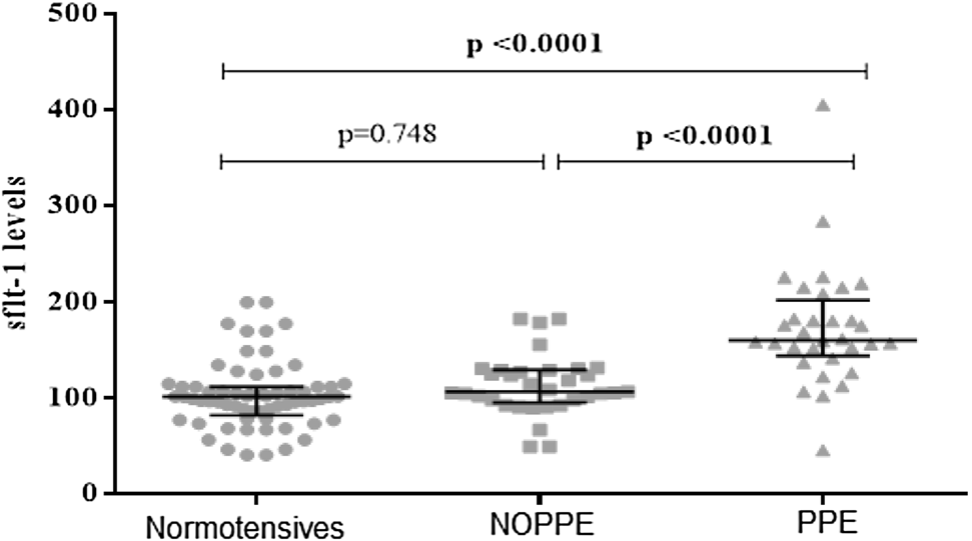
Serum levels of sFlt-1 among the participants. The levels of sFlt-1 did not differ between the NOPPE women and the normotensive controls but were higher in the PPE women compared to the NOPPE and normotensive women.

Endothelial function is regulated by sVCAM-1, and marked elevation of the serum levels of sVCAM-1 indicates a state of compromised endothelial function^41–43^ as a result of atherosclerotic plaques^44,45^ caused by dyslipidemia^46^. In this study, sVCAM-1 levels were significantly elevated in the NOPPE and PPE women, compared to the normotensive controls; but were significantly higher in the PPE than in the NOPPE women (Fig. 1). This finding suggests that endothelial dysfunction may be more severe in PPE, as compared to NOPPE. The observed higher levels of TC, TG (Table 3) and sFlt-1 (Fig. 2) in the PPE women from our study affirms our assertion.

Assessment of the extent of liver damage is partly achieved through the measurement of AST and ALT levels in the serum. Although, low in specificity, AST and ALT are the most widely used parameters in assessing liver damage, especially in resource-limited environments. We verified and compared serum levels of AST and ALT in the NOPPE and PPE women because antepartum PE is associated with multi-organ damage, including the liver. The observed higher levels of AST and ALT in the PPE compared to the NOPPE and normotensives (Table 3) may indicate that liver damage is more severe in the PPE than in the NOPPE. Furthermore, our findings suggests that in predicting the occurrence of NOPPE, high AST and ALT levels may provide the best diagnostic reliability and may be useful especially in areas where high-level and cutting-edge methods are not available.

## Study limitations

The retrospective prevalence study was based on a single study site. Multiple sites would be useful to provide a regional or national prevalence of postpartum PE.

## Conclusion

The prevalence of PPE was 11.9%. Sedentary lifestyle was a common occurrence among these women (NOPPE and PPE). Dyslipidaemia, hypomagnesaemia, endothelial dysfunction and, possibly, liver impairment were more prevalent and severe in the PPE as compared to the NOPPE women. These findings indicate that NOPPE and PPE are different pathological conditions that require different clinical attention. Combined assessment of glucose, lipids and liver enzymes would be instructive in identifying pregnant woman at risk of developing NOPPE. Our results emphasize that there is risk for developing PE, even in the postpartum period. We emphasize the need for intensified education on antepartum PE and postpartum PE, and the integration of exercise therapies into the routine obstetric prescriptions that form the basis of antenatal care, especially in sub-Saharan Africa.

## Data Availability

All dataset used for the study are within the manuscript.
